# Design and Validation of a Scale for Measuring Well-Being of Children in Lockdown (WCL)

**DOI:** 10.3389/fpsyg.2020.02225

**Published:** 2020-09-18

**Authors:** Naiara Berasategi, Nahia Idoiaga, Maria Dosil, Amaia Eiguren

**Affiliations:** ^1^Department of Didactics and School Organisation, University of the Basque Country - UPV/EHU, Bilbao, Spain; ^2^Department of Evolutionary and Educational Psychology, University of the Basque Country - UPV/EHU, Bilbao, Spain; ^3^Department of Research and Diagnostic Methods in Education, University of the Basque Country - UPV/EHU, Bilbao, Spain

**Keywords:** children, well-being, scale, validation, lockdown, COVID-19

## Abstract

The objective of this study was to create and validate an instrument to measure the well-being of children in lockdown. As a response to the COVID-19 pandemic, and in the interest of maintaining social distancing, millions of people have been confined to their homes, including children, who have been withdrawn from school and barely able to leave their homes. Thus, it would be useful to evaluate, from a holistic perspective, the well-being of children under these challenging circumstances. The participants were 1,046 children, 48.7% of which were boys and 50.7% girls, recruited in the Basque Country (Northern Spain). The scale was answered by their parents. The survey, entitled “Well-being of Children in Lockdown” (WCL), is composed of six subscales: Emotions, Playful and creative activities, Education, Addictions, Routine, and Physical Activity. Exploratory factor analyses indicate that all the reliability indices were acceptable. The survey demonstrated adequate reliability (alpha = 0.804). We were thus able to confirm the validity of this simple instrument for evaluating the well-being of children aged between 4 and 12 years in lockdown situations. The WCL can be regarded as a useful tool to evaluate the well-being of children in lockdown situations.

## Introduction

The new coronavirus (COVID-19) epidemic has created an unprecedented threat to global health. The outbreak first emerged in late December 2019 when clusters of pneumonia cases of unknown etiology were found in China. Since then, the number of cases has continued to escalate exponentially, firstly within China and then worldwide. On the 30th of January 2020, the World Health Organization (WHO) declared the COVID-19 outbreak a public health emergency of international concern, and on the 11th of March 2020, it was declared a pandemic (World Health Organization [WHO], 2020).

Children represent a small percentage of COVID-19 cases (Hamzelou, 2020; Pavone et al., 2020) with most infected infants being asymptomatic (Cai et al., 2020) or presenting only mild clinical manifestations (Jiao et al., 2020). However, children are not impervious to the dramatic impact of the COVID-19 epidemic. In fact, it has been pointed out that, due to the mild symptomatology shown by children, they could play a prominent role in spreading COVID-19. As a consequence, in most countries of the world schools have been ordered to close (United Nations Educational, Scientific and Cultural Organization [UNESCO], 2020) and children, like the rest of the population, have been confined to their homes. Nonetheless, each country has its own set of rules and guidelines in relation to the lockdown. Thus, while, in some countries, children can go out for sports or walks, in other countries such activities have been prohibited (García, 2020). Spain is one of the countries in which children have faced the most stringent lockdown regulations, since from March 14th to 26th April (a period of 6 weeks) children had been completely banned from leaving their homes. Since then, a slight easing of the lockdown measures has meant that from 26th April onward, children have been allowed outside, but only for 1 h each day and they must remain within close proximity of their homes (Lucas, 2020).

Furthermore, this reality does not affect only Spain; countries around the world have been affected among other Latin countries like Italy in Europe (Pisano et al., 2020). Besides, it has been stated that for example in Latin America children are the hidden victims of COVID-19 crisis (Catalán, 2020; SOS Children’s Villages International, 2020; TRT Español, 2020; United Nations International Children’s Emergency Fund [UNICEF], 2020).

Pediatricians, psychologists, and educators have all warned of the threats that this lockdown could have for the well-being of children, from both physical and emotional perspectives (Grechyna, 2020; Jiloha, 2020). Moreover, international researchers are already studying these consequences from multiple perspectives. At a physical level, research conducted in China has found that during lockdown, 3- to 18-year-old children are physically less active, have much longer screen time, show irregular sleep patterns, and eat less favorable diets, all of which is resulting in weight gain and a loss of cardiorespiratory fitness (Jiao et al., 2020; Jiloha, 2020; Wang et al., 2020). In fact, this dramatic reduction in physical activity and insufficient exposure to sunlight as a result of being forced to remain at home have been highlighted as some of the most visible consequences of this lockdown situation (Lippi et al., 2020).

From an emotional perspective, research carried out in China has found that lockdown is generating feelings of fear, worry, sadness, loneliness, or stress among children from 3 to 18 years (Jiao et al., 2020; Jiloha, 2020; Leung et al., 2020; Qiu et al., 2020). Added to this are observations of clinginess, distraction, irritability, and an apparent fear of asking questions about the pandemic (Wang et al., 2020). In a similar vein, a research study in Italy with children aged between 4 and 10 years has found that, during this lockdown, children are showing fears that they had never expressed before, along with increased irritability, nervousness, intolerance to rules, whims and excessive demands, mood changes, and sleep problems (Pisano et al., 2020).

**FIGURE 1:**
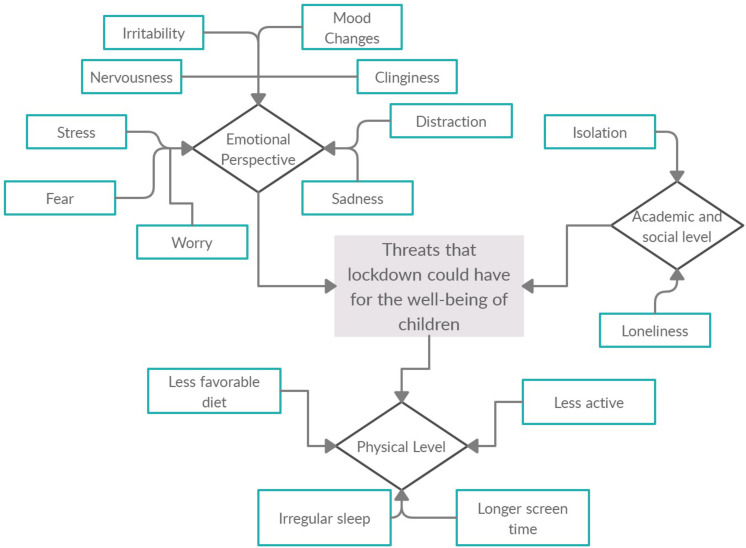
Summary of threats that lockdown could have for the well-being of children.

At academic and social levels, social isolation and lockdown means that children from preschool, primary school, and secondary school may not be at school for a prolonged period of time (Jiao et al., 2020) and their social interactions will be limited, thereby reducing dramatically the possibilities of socializing and playing with peers (Wang et al., 2020), which could only serve to exacerbate the sense of loneliness felt during lockdown (Jiao et al., 2020; Okruszek et al., 2020; Singh and Singh, 2020). Several researchers have noted that these disruptions could also have long-term consequences for the affected groups and that, for the most vulnerable members of the population, existing inequalities are likely to become even more evident (Armitage and Nellums, 2020; Burgess and Sievertsen, 2020) [see Figure 1].

Thus, children are not unaffected by the dramatic impact of the COVID-19 epidemic, and their well-being in this situation is likely to be influenced in various ways.

The concept of well-being is highly variable and has been studied across a wide range of disciplines, age groups, cultures, communities, and countries, resulting in a wide range of definitions (Pollard and Lee, 2003). In fact, this debate has resulted in well-being becoming a field of research in its own right (Amerijckx and Humblet, 2013). From a holistic view, well-being has been defined as “a multidimensional construct incorporating mental/psychological, physical and social dimensions” (Columbo, 1986, p. 288). In the same vein, in reference to health, and according to the World Health Organization [WHO] (1946) “Health is a state of complete physical, mental and social well-being and not merely the absence of disease or infirmity” (p. 100). Some authors agree that children’s well-being cannot be represented by a single domain or indicator, as their lives are lived in terms of multiple domains and each domain has an impact on their well-being (Ben-Arieh et al., 2001; Bradshaw and Mayhew, 2005; Hanafin and Brooks, 2005a,b; Land et al., 2007; Domínguez-Serrano et al., 2019; Fattore et al., 2019; Migliorini et al., 2019). For example, Brandshaw and Richardson (2009) have argued that children’s well-being should be represented in terms of seven domains: (1) health; (2) subjective well-being; (3) personal relationships; (4) material resources; (5) education; (6) behaviors and risks; and (7) housing and environment. Indeed, the most recent reference may be the “Index of Child and Youth Well-Being” by Land et al. (2007). This index was created to measure changes in child well-being in the United States and can serve as an example for a system of analysis based on outcome indicators. Specifically, that system is based on the results of subjective well-being studies that identify content areas that occur over and over again—for guidance in the selection of domains of well-being and statistical indicators within those domains. Concretely, those domains are family economic well-being, health, safety/behavioral concerns, educational attainment (productive activity), community connectedness (participation in schooling or work institutions), social relationships (with family and peers), and emotional/spiritual well-being.

Although challenging, there are both theoretical and practical reasons for approaching well-being as a multidimensional construct across life domains (Huppert and So, 2013) and while the integration of various dimensions has been defined as fundamental to achieving positive well-being throughout the lifespan (Zaff et al., 2003), relatively little research has been dedicated to the cognitive, emotional, physical, and social aspects of children’s well-being (Ajdukovic and Ajdukovic, 1993; Househnecht and Sastry, 1996; McCormick et al., 1996; Evans et al., 1998; Qin et al., 2020). Therefore, there is a relatively small body of national data for the indicators used to track child health and well-being, which all countries have agreed to collect (Clark, 2020).

Likewise, there are few standardized methods for assessing well-being in childhood. The majority of researchers who have analyzed child well-being used multiple separate measures of pre-assumed indicators in an effort to capture a more complete assessment of the state of the child’s well-being (Pollard and Lee, 2003). Pollard and Lee (2003) conducted a systematic review of the literature on child well-being by searching five databases to assess the current state of child well-being research to address the following questions: (1) How do we define the well-being of children? (2) What are the domains of children’s well-being? (3) What are the indicators of children’s well-being? and (4) How do we measure the well-being of children? In relation to the scales measuring children’s well-being, these authors conclude that there is an inconsistent use of definitions, indicators, and measures of well-being, which has created a confusing and contradictory research base.

Among those scales that analyze the well-being of children from a holistic perspective, taking into account the physical, social, and psychological dimensions (among others), the Perceived Competence Scale for Children (Harter, 1982) is noteworthy. Designed to measure 8–13-year-old children’s perceptions of their competence and self-adequacy, this scale considers their cognitive competence, peer relationships, scholastic performance, physical skills/competence, and global self-worth. In a similar vein, The Battelle Developmental Inventory Screening Test (Newborg et al., 1988), which was designed as a tool for the screening, diagnosis, and evaluation of early development, takes into account the self-concept, affect, coping, adult interaction, peer interaction, social role, personal responsibility, eating, dressing, attention, toileting, receptive and expressive communication, academic skills, memory, reasoning, cognitive development, perceptual motor, locomotion, muscle control, and body coordination from birth to 8 years. Finally, Castilla-Peón (2014) used a method of direct questioning to evaluate the well-being of children aged between 11 and 15 years, in relation to family, school, play, growth, development, friends, and peers.

Another international study—which did not take into account emotional and physical domains— was carried out by The Children’s Worlds, The International Survey of Children’s Well-Being (ISCWeB), which is a worldwide research survey of children’s subjective well-being. The questionnaire consists of eight life domains: the home and the people they live with, money and things they have, relationships with friends and other people, the area where they live, school, health, time management, and leisure time and self. Indeed, there are also recent studies that evaluate the well-being of children with chronic diseases and/or with cognitive, motor, and social disorders as autism (Salomone et al., 2018); refugee children (Baker et al., 2019); physical limitations, as child’s spinal cord injury (January et al., 2019); or adverse behavioral of school-age children relating to sleep duration (James and Hale, 2017). This reinforces that it is more and more relevant to investigate the general well-being of the child, taking into account that previous illnesses may further influence his general condition.

In addition, there is no single scale to measure the well-being of children in lockdown that could help to identify the physical, psychological, social, and academic consequences of this situation. Such information is critical if we are to establish the actions that can be taken to mitigate the negative effects of confinement and improve the well-being of children. Moreover, at all life stages, but even more so in childhood, the biological, psychological, and social processes are merged into a network of intimate interactions that make it impossible to conduct studies or analyses from a unidirectional approach (Villaroel, 2012). Thus, given that in lockdown situations there are threats to children’s health and well-being that originate from multiple levels or dimensions, a deliberately multidimensional approach is required to safeguard the well-being of children. Therefore, the main objective of this study was to create and validate an easy-to-use survey that is capable of measuring—from a holistic perspective—the well-being of children in a situation of lockdown. Based on the theoretical framework described above, it is hypothesized that the scale that measures the well-being of children will have several factors or domains that converge in adequate psychometric properties of the overall well-being of children in lockdown situation.

## Materials and Methods

### Participants

A total of 1046 children participated in this study. Of these, 48.7% were boys (*n* = 505) and 50.7%% girls (*n* = 530), aged between 2 and 14 years (*M* = 6.43; *SD* = 2.95). 74.5% of those children said they have another brother or sisters (*n* = 779), and 25.5% said no (*n* = 267). 2.5% had special educational needs (*n* = 26), and 97.5% did not have educational special needs (*n* = 1020). Regarding exterior spaces that they have in their home, 36.6% said that they did not have an exterior space (*n* = 383) and 63.4% said no (*n* = 663). Lastly, regarding the socioeconomic status of their parents the majority, 72.86% were from a high class (*n* = 639), 24.74% were of low status (*n* = 217), and 2.4% were of high socioeconomic status. All the information about the children was gathered through their parents, as they were the ones who answered the scale.

All participants were recruited from the Autonomous Community of the Basque Country in the North of Spain.

### Instrument

The survey entitled “Well-being of Children in Lockdown” (WCL) was used. The preliminary version of the survey was drawn up by a group of university professors experienced in Childhood and Infectious Diseases. In order to create each of the items, preliminary qualitative information was collected with regard to the situation of children in lockdown (Idoiaga et al., 2020; Idoiaga Mondragon et al., 2020). Furthermore, we took as a reference all of the surveys and works that include the various dimensions and indicators used for children’s well-being (cited in the section “Introduction”).

The preliminary version included a total of 26 items, 3 items in the “Academic” dimension, 4 items in the “Routine” dimension, 2 items in the “Physical activity” dimension, 5 items in the “Emotions” dimension, 4 items in the “Addiction” dimension, and 4 items in the “Playful and creative activities” dimension. A 4-point Likert scale response format was chosen, ranging from 1 = strongly disagree to 4 = strongly agree. In order to ensure both the validity of the content and applicability of the instrument, this initial version was subject to a two-step refinement process:

(1) *Expert consideration*. In order to ensure the validity of its content, the first version of the survey was submitted to a panel of 4 experts in research and childhood education. Using a purpose-designed table, the experts had to evaluate the dimension corresponding to each item according to its content, as well as the degree of precision and clarity. They were also invited to make suggestions for improving the draft. The only items kept were those considered by 3 out of the 4 experts to be well written and those for which all experts agreed on their inclusion within a given dimension.
(2) *Pilot study*. A total of 65 parents of children aged between 2 and 14 years participated with a view to modifying and/or eliminating the most problematic items in terms of understanding or those that contained errors in their formulation. Three items were eliminated from each dimension, meaning that the final version (Table 1) was reduced to 22 items.

**TABLE 1:** Survey on the well-being of children in lockdown.

(1)	Your child has been sent materials, assignments, and homework by your school (¿Has recibido recursos, propuestas o deberes enviados desde la escuela?)	1	2	3	4
(2)	Your child spends enough time on your schoolwork during the day (¿Dedica un tiempo adecuado al trabajo escolar durante el día?)	1	2	3	4
(3)	Your child has an agreed routine and you try to stick to it (¿Tiene una rutina establecida e intentamos mantenerla?)	1	2	3	4
(4)	Your child usually has breakfast, lunch, and dinner at the same time each day (¿Desayuna, come y cena normalmente a la misma hora?)	1	2	3	4
(5)	Your child gets enough physical exercise during the day (¿Hace suficiente ejercicio físico durante el día?)	1	2	3	4
(6)	Your child moves his/her body enough (¿Mueve su cuerpo suficiente?)	1	2	3	4
(7)	Your child has healthy sleeping habits (¿Tiene unos hábitos saludables de sueño?)	1	2	3	4
(8)	Your child cries more than usual (¿Llora más de lo normal?)	1	2	3	4
(9)	Your child feels more nervous than usual (¿Está más nervioso/a que lo habitual?)	1	2	3	4
(10)	You get angry more than usual (¿Se enfada más de lo habitual?)	1	2	3	4
(11)	Your child feels sadder than usual (¿Está más triste de lo habitual?)	1	2	3	4
(12)	Your child is happy (¿Está contento?)	1	2	3	4
(13)	Your child is eating a well-balanced diet (¿Lleva una dieta equilibrada?)	1	2	3	4
(14)	Your child is eating more than usual during lockdown (¿Come más de lo normal en esta situación de confinamiento?)	1	2	3	4
(15)	Your child is eating more treats (e.g., cookies, chocolate, and chips) during lockdown (¿Come más chucherías (Galletas, chocolate, patatas, etc.) o comida rápida en esta situación de confinamiento?)	1	2	3	4
(16)	Your child is overusing new technology (¿Está abusando de las nuevas tecnologías?)	1	2	3	4
(17)	Your child is watching too many TV programs, cartoons, or movies (¿Está abusando de ver la tele, dibujos o películas)	1	2	3	4
(18)	Your child is taking part in creative activities (e.g., theater, music, and art) (¿Realiza actividades para trabajar la creatividad (Teatro, música, arte…)?	1	2	3	4
(19)	Your child plays different games throughout the day (¿Juega a diferentes cosas a lo largo del día?)	1	2	3	4
(20)	Your child works on school projects with your family throughout the day (¿Realizamos a lo largo del día actividades escolares en familia?)	1	2	3	4
(21)	Your child does leisure activities with your family throughout the day (¿Realizamos a lo largo del día actividades lúdicas en familia?)	1	2	3	4
(22)	Your child plays with your family throughout the day (¿Jugamos en familia a lo largo del día?)	1	2	3	4

*Emotions: 8, 9, 10, 11, 12; activities: 18, 19, 21, 22; academic: 1, 2, 20; addiction: 14, 15, 16, 17; routine: 3, 4, 7, 13; physical activity: 5, 6.*

The final scale consisted of the following 6 dimensions: Emotions (5 items), Playful and creative activities (4 items), Academic (3 items), Addictions (4 items), Routine (4 items), and Physical activity (2 items). The items included in the first dimension are related to emotions, those in the second dimension are related to playful or creative activities, the third dimension consists of items related to academic issues, the items of the fourth dimension are related to habits of overuse (technology or eating habits), the fifth dimension is concerned with daily routines (the maintenance of a daily schedule, e.g., eating and sleeping habits), and finally, the sixth dimension contains items related to physical activity. The participants were required to respond on a 4-point Likert-type scale ranging from strongly agree (4) to strongly disagree (1). Items 8, 9, 10, 11, 14, 15, 16, and 17 were recorded, since they had been formulated in a negative way. The various subscales were shown to have adequate values of internal consistency (Cronbach’s alpha > 0.60).

### Procedure

The project was approved by the Ethics Committee of the Basque Country University of [M10/2020/055]. The data were collected during the period of confinement from March 14th to April 22nd. This study was conducted ethically according to the principles in line of the Declaration of Helsinki.

In order to recruit the participants, all the centers registered in the database of the Department of Education of the Basque Government were considered, and the schools were asked to forward these questionnaires to the families of the pupils. Both the data of the sample and the consent for participation in the study were collected with the help of Google online forms. Family members were informed of the research study by e-mail. In the same questionnaire, it was explained that participation in the study was voluntary and anonymous. Moreover, the parents or legal guardians of the children gave written consent for two phases of this research. The questionnaire was filled by the parents, and it takes around 5 min to fill out. Consent was given to, first, analyze the data and, second, to make the data public in scientific articles while respecting anonymity. A total of 30 questionnaires were excluded for not giving consent for this second phase.

### Statistical Analysis

All of the data were analyzed using the statistics program SPSS version 24.0 (IBM, Chicago, IL, United States). For the purpose of comparing the proposed measurement scale, exploratory factor analysis was carried out to identify the number and composition of the common factors (latent variables) necessary to explain the common variance of all items analyzed and to thus validate the scale.

The calculation that determines the desired sample for this type of research is calculated through a statistical platform.

Univariate statistics (mean and standard deviation) were calculated for each item, and factor analysis was carried out to analyze the dimensionality of the scale.

In each dimension, an independent calculation of the partial item test was made to estimate the item discrimination rate. Cronbach’s alpha coefficient was used to calculate reliability. Confirmatory factor analysis was conducted in an attempt to confirm the factor structure obtained.

The significance value indicating that the association is statistically significant has been arbitrarily selected and by consensus is considered to be 0.05. A 95% confidence carries an implicit *p <* 0.05 (Fisher, 1971). For the calculation of the magnitude of the results, the size of the effect was calculated by Lenhard and Lenhard (2016) and interpreted by Cohen (1988).

## Results

### Exploratory Factor Analyses

Once the exploratory factor analysis had been carried out, 6 factors were rotated with 26 items and 4 were eliminated to obtain a load of less than 0.30. Both the Bartlett statistic [8325.42(df = 231; *P* < 0.000)] and the Kaiser–Meyer–Olkin test (KMO) = 0.799 show adequate fit of the data for subsequent factor analysis. The six factors extracted explain 62.7% of the total variance. The first factor explains 22.2% of the variance, the second factor 12.29% of the variance, the third factor 9.23% of the variance, the fourth factor 6.81% of the variance, the fifth factor 6.40% of the variance, and the sixth factor 5.82% of the variance. These data show an excellent fit of a six-dimensional structure for these items (García et al., 1998) [see Table 1].

The first factor, termed “Emotions,” contains a series of items (8, 9, 10, 11, and 12) that explore emotional aspects. The second factor “Playful and creative activities” contains items (18, 19, 21, and 22) related to playful and creative activities. The third factor, “Academic,” consists of items (1, 2, and 20) referring to educational aspects. The fourth factor, “Addiction,” consists of a series of items (14, 15, 16, and 17) looking at the overuse of new technology, or overeating.

Items of the fifth factor (3, 4, 7, and 13) are concerned with the daily routine, referring to aspects such as timetable, diet, and sleeping habits. Finally, the sixth factor asks about physical activity (items 5 and 6). The estimated reliability coefficients were 0.872 for the first factor, 0.783 for the second, 0.696 for the third, 0.627 for the fourth, 0.646 for the fifth, and 0.847 for the sixth factor. The reliability of the entire scale was 0.804.

For the same scale, Table 2 indicates the main statistics for the items that make up the scale (mean and standard deviation). It is clear that the items in intermediate positions near the mid-point of the cutoff are Item 5, related to the amount of physical activity during the day (*M* = 2.44); the items of Factor 3 (Education); Item 2, which is related to how much time they spend on school tasks (*M* = 2.44); and Item 10, related to how much time they spend on school activities with their families (*M* = 2.54). A similar score was obtained for Item 17, related to new technology (*M* = 2.5).

**TABLE 2:** Mean, standard deviation, rotated factor matrix, and reliability analysis of variables and factors.

	*M*	*SD*	F1	F2	F3	F4	F5	F6
Item 1.	2.92	0,85	0.029	−0.044	0.805	−0.018	−0.022	−0.024
Item 2.	2.55	0,84	−0.024	−0.125	0.825	−0.012	0.070	0.020
Item 3.	2.95	0,69	−0.056	0.133	0.184	0.122	0.641	0.119
Item 4.	3.35	0,65	0.062	0.054	−0.041	−0.022	0.815	0.020
Item 5.	2.44	0,7	0.064	0.130	0.004	0.147	0.092	0.886
Item 6.	2.61	0,72	0.160	0.173	−0.050	0.064	0.096	0.875
Item 7.	3.28	0,66	0.180	0.081	−0.014	0.050	0.709	0.019
Item 8.	3.18	0,89	0.799	−0.134	0.158	0.057	0.070	−0.015
Item 9.	2.9	0,94	0.871	0.027	−0.006	0.169	0.011	0.041
Item 10.	2.75	0,97	0.874	0.044	0.041	0.157	0.034	0.050
Item 11.	3.23	0,84	0.800	0.094	−0.084	0.148	0.057	0.112
Item 12.	3.07	0,67	0.591	0.353	−0.151	0.132	0.161	0.149
Item 13.	3.38	0,57	0.038	0.202	−0.018	0.348	0.486	0.072
Item 14.	3.06	0,86	0.214	−0.033	0.097	0.669	0.083	−0.067
Item 15.	3.12	0,74	0.185	−0.027	0.056	0.745	0.120	−0.010
Item 16.	2.48	0,84	0.035	0.332	−0.161	0.586	0.046	0.223
Item 17.	2.5	0,79	0.112	0.175	−0.041	0.579	0.033	0.203
Item 18	2.64	0,84	−0.052	0.628	0.145	0.024	0.023	0.201
Item 19.	3.08	0,71	0.079	0.717	−0.003	0.110	0.107	0.196
Item 20.	2.54	0,87	0.016	0.244	0.701	0.044	0.042	−0.043
Item 21.	2.92	0,67	0.069	0.834	0.003	0.086	0.150	−0.014
Item 22.	2.93	0,71	0.064	0.832	−0.074	0.083	0.136	−0.018

In contrast, higher scores are obtained for the items corresponding to Factor 5 (Routine), particularly on Item 13, which is related to whether they eat a well-balanced diet (*M* = 3.38), Item 4, which asks if they have breakfast, lunch and dinner at the same time each day (*M* = 3.35), and Item 7, which asks about healthy sleeping habits (*M* = 3.28).

### Bivariate Correlations and Effect Size

Table 3 shows the correlations between all the elements of the study. It is evident that the highest correlations can be observed between the elements measuring the same dimension, showing a larger size of the effect. Effect sizes vary (from no effect to a large effect).

**TABLE 3:** Bivariate correlations between all elements of the study and effect size.

	1	2	3	4	5	6	7	8	9	10	11	12	13	14	15	16	17	18	19	20	21	22
(1)	−																					
(2)	0.517** (0.267)	−																				
(3)	0.062* (0.001)	0.167** (0.003)	−																			
(4)	−0.027 (−0.001)	−0.005 (−0.001)	0.382** (0.15)	−																		
(5)	−0.0036 (−0.001)	−0.016 (−0.001)	0.169** (0.03)	0.100** (0.001)	−																	
(6)	−0.056 (−0.001)	−0.067* (−0.001)	0.141** (0.02)	0.118** (0.02)	0.735** (0.54)	−																
(7)	−0.006 (−0.001)	0.031 (0.001)	0.262** (0.07)	0.430** (0.20)	0.107** (0.02)	0.133** (0.02)	−															
(8)	0.117** (0.02)	0.122** (0.002)	0.039	0.072* (0.001)	0.037 (0.001)	0.099** (0.001)	0.174** (0.03)	−														
(9)	0 (0)	−0.023 (−0.001)	0.004 (−0.001)	0.06 (0.001)	0.134** (0.02)	0.184** (0.03)	0.156** (0.02)	0.641** (0.41)	−													
(10)	0.023 (0.001)	0.024 (0.001)	0.069* (0.001)	0.089** (0.001)	0.143** (0.02)	0.182** (0.03)	0.163** (0.03)	0.678** (0.46)	0.781** (0.61)	−												
(11)	−0.042 (−0.001)	−0.093** (−0.002)	0.057 (0.001)	0.107** (0.01)	0.170** (0.03)	0.241** (0.06)	0.175** (0.03)	0.523** (0.27)	0.648** (0.42)	0.633** (0.40)	−											
(12)	−0.085** (−0.001)	−0.160** (−0.03)	0.090** (0.001)	0.185** (0.03)	0.222** (0.05)	0.304** (0.09)	0.220** (0.05)	0.273** (0.07)	0.475** (0.22)	0.477** (0.23)	0.586** (0.34)	−										
(13)	0.014 (0.001)	−0.002 (−0.001)	0.265** (0.07)	0.262** (0.07)	0.190** (0.03)	0.183** (0.04)	0.272** (0.07)	0.06 (0.001)	0.151** (0.02)	0.099** (0.001)	0.125** (0.01)	0.271** (0.07)	−									
(14)	0.091** (0.001)	0.011 (−0.001)	0.108** (0.002)	0.094** (0.001)	0.099** (0.001)	0.094** (0.001)	0.126** (0.02)	0.186** (0.03)	0.277** (0.07)	0.269** (0.07)	0.240** (0.02)	0.201** (0.04)	0.192** (0.04)	−								
(15)	0.02 (0.001)	0.02 (−0.001)	0.124** (0.02)	0.108** (0.02)	0.163** (0.03)	0.112** (0.02)	0.116** (0.01)	0.206** (0.04)	0.268** (0.07)	0.249** (0.05)	0.265** (0.06)	0.231** (0.05)	0.287** (0.08)	0.447**	−							
(16)	−0.129** (−0.02)	−0.132** (−0.02)	0.162** (0.03)	0.093** (0.001)	0.261** (0.07)	0.250** (0.06)	0.164** (0.02)	0.016 (0.001)	0.158** (0.03)	0.179** (0.03)	0.197** (0.04)	0.274** (0.06)	0.218** (0.05)	0.188**	0.256**	−						
(17)	−0.083** (−0.001)	0.004 (−0.001)	0.146** (0.02)	0.071* (0.001)	0.231** (0.05)	0.202** (0.04)	0.166** (0.03)	0.160** (0.03)	0.198** (0.04)	0.234** (0.05)	0.212** (0.04)	0.181** (0.03)	0.163** (0.02)	0.174** (0.03)	0.241** (0.06)	0.487** (0.24)	−					
(18)	0.047 (0.001)	0.036 (0.001)	0.154** (0.02)	0.080** (0.001)	0.193** (0.03)	0.221** (0.05)	0.068* (0.001)	−0.078* (−0.001)	0 (0)	0.009 (0.001)	0.064* (0.001)	0.191** (0.04)	0.175** (0.03)	0.023 (0.001)	0.027 (0.001)	0.224** (0.05)	0.128** (0.02)	−				
(19)	−0.025 (−0.001)	−0.083** (−0.001)	0.161** (0.03)	0.152** (0.02)	0.266** (0.07)	0.296** (0.09)	0.179** (0.03)	0.005 (0.001)	0.113** (0.01)	0.121** (0.2)	0.150** (0.02)	0.343** (0.12)	0.227** (0.05)	0.100** (0.01)	0.118** (0.01)	0.321** (0.10)	0.199** (0.03)	0.477** (0.23)	–			
(20)	0.381** (0.145)	0.406** (0.165)	0.130** (0.02)	0.061* (0.001)	0.027 (0.001)	−0.006 (−0.001)	0.080** (0.001)	0.044 (0.001)	0.014 (0.001)	0.037 (0.001)	0.011 (0.001)	0.055 (0.001)	0.015 (0.001)	0.067* (0.001)	0.067* (0.001)	0.031 (0.001)	0.047 (0.001)	0.148** (0.02)	0.122** (0.02)	−		
(21)	−0.03 (−0.001)	−0.073* (−0.001)	0.224** (0.05)	0.149** (0.02)	0.194** (0.03)	0.205** (0.04)	0.161** (0.03)	−0.009 (−0.001)	0.107** (0.02)	0.124** (0.02)	0.123** (0.01)	0.296** (0.07)	0.263** (0.06)	0.078* (0.001)	0.107** (0.01)	0.266** (0.06)	0.193** (0.03)	0.366** (0.13)	0.481** (0.23)	0.166** (0.03)	–	
(22)	−0.096** (−0.002)	−0.126** (−0.02)	0.193** (0.04)	0.151** (0.02)	0.184** (0.03)	0.212** (0.04)	0.156** (0.02)	−0.019 (−0.001)	0.097** (0.001)	0.115** (0.01)	0.129** (0.01)	0.282** (0.07)	0.242** (0.05)	0.072* (0.001)	0.097** (0.001)	0.274** (0.07)	0.199** (0.03)	0.337** (0.11)	0.495** (0.25)	0.128** (0.02)	0.775** (0.60)	–

***p <0.0000, **p <0.001, *p <0.01.

## Discussion and Conclusion

In order to address our proposed objective, the definitive “Well-being of Children in Lockdown Situations” (WCLS) scale was submitted to the following statistical tests: exploratory factor analysis, reliability analysis using Cronbach’s alpha, and bivariate correlations. The exploratory factor analyses revealed that the reliability indices were acceptable in all cases, while Cronbach’s alpha values were found to be above the minimum recommended value of 0.80 (Nunnally, 1978), with the total alpha for the scale standing at 0.80, meaning that the reliability of the measurements can be considered adequate. Further, it has been established that the factor structure of the scale is compatible with the predicted factors and reconfirms their weight and level of confidence. Taken together, the results of all of the analyses indicate that the WCL has adequate psychometric properties.

This methodological process was analyzed with the objective of obtaining a reliable and valid research instrument for gathering information on the well-being of children in a lockdown situation. In the light of the comments made in the results section, we can confirm an adequate fit of the data to the dimensional structure of the items making up the scale. We therefore consider that this could be a highly useful instrument for evaluating the well-being of children amid these challenging circumstances. In particular, this scale could help to identify how children are feeling, along with their well-being needs, since this knowledge will be of vital importance if we are to manage this health crisis in the best possible way. The size of the scale (22 items) makes this an easy to use instrument, while its extension—enabling its use at different stages of education—makes it highly useful.

In order to safeguard the immediate future of all children, a holistic strategy is needed in response to the uncertainty that surrounds them as a result of COVID-19. Therefore, it should be in the interests of all stakeholders—from governments and researchers to parents—to protect the physical, psychological, social, and academic well-being of children in this current public health crisis. In light of all the issues that have already been mentioned, we consider that the WCL scale represents an advance in the study of well-being. In particular, this scale will be useful for both the present health crisis and those that might arise in the future, particularly since there is currently no instrument that measures the well-being of children during a lockdown situation.

Overall, this study shows that WCL-S has satisfactory psychometric properties. The availability of a reliable and shortened tool for measuring the well-being of children from a holistic way in a lockdown situation is important for two main reasons. First, it could help to explain who has the situation where children attend to different aspects of their well-being (emotional, social, academic, and physical), and second, it could be beneficial from a research perspective, for example given detailed information of children’s well-being to know who to prevent and redirect the situation taking into account the lacks that could be in the different dimensions analyzed from a holistic view.

In terms of expanding the findings and overcoming some of the limitations of this study, three general directions for future research could be recommended. First, to obtain further evidence of the social, cultural, or religious aspects that can influence the results, other specific questions or scales could be added in order to analyze the data taking into account those aspects. Also, aspects in relation with parents’ situation (emotional, social, or economic) will be interesting to collect in order to analyze what can influence the well-being of children. Second, to study the role that religion plays in lockdown situations, it would be interesting to analyze how religious families can influence the well-being of children. Third, attempting to generalize the findings of this study to related interventions, it could be useful to examine the results of the well-being of children in other samples.

## Data Availability Statement

The raw data supporting the conclusions of this article will be made available by the authors, without undue reservation, to any qualified researcher.

## Ethics Statement

The studies involving human participants were reviewed and approved by The Committee of Ethics for Research related to Human Beings of the University of the Basque Country (CEISH). Written informed consent to participate in this study was provided by the participants’ legal guardian/next of kin.

## Author Contributions

All authors listed have made a substantial, direct and intellectual contribution to the work, and approved it for publication.

## Conflict of Interest

The authors declare that the research was conducted in the absence of any commercial or financial relationships that could be construed as a potential conflict of interest.
